# Down-Regulation of CXCL12/CXCR4 Expression Alleviates Ischemia-Reperfusion-Induced Inflammatory Pain via Inhibiting Glial TLR_4_ Activation in the Spinal Cord

**DOI:** 10.1371/journal.pone.0163807

**Published:** 2016-10-19

**Authors:** Xiao-Qian Li, Zai-Li Zhang, Wen-Fei Tan, Xi-Jia Sun, Hong Ma

**Affiliations:** Department of Anesthesiology, First Affiliated Hospital, China Medical University, Shenyang 110001, Liaoning, China; Katholieke Universiteit Leuven Rega Institute for Medical Research, BELGIUM

## Abstract

Toll-like receptor 4 (TLR_4_) is important for the pathogenesis of inflammatory reactions and the promotion of pain processing after ischemia/reperfusion (IR) in spinal cord. Recently, C-X-C chemokine ligand 12 (CXCL12) and its receptor, C-X-C chemokine receptor 4 (CXCR4), were demonstrated to be simultaneously critical for inflammatory reactions, thereby facilitating glial activation. However, whether CXCL12/CXCR4 expression can contribute to IR-induced inflammatory pain via spinal TLR_4_ remained unclear. A rat model was established by 8 min of aortic arch occlusion. The effects of CXCL12/CXCR4 expression and TLR_4_ activation on inflammatory hyperalgesia were investigated by pretreatments with CXCL12-neutralizing antibody, CXCR4 antagonist (AMD3100) and TLR_4_ antagonist (TAK-242) for 5 consecutive days before surgery. The results indicated that IR induced significant and sustained inflammatory pain, observed as decreases in paw withdrawal threshold (PWT) and paw withdrawal latency (PWL), throughout the post-injury period. The increased levels of TLR_4_ and proinflammatory chemokine CXCL12, as well as its receptor, CXCR4, were closely correlated with the PWT and PWL trends. Double immunostaining further suggested that TLR_4_, which is mainly expressed on astrocytes and microglia, was closely co-localized with CXCL12 and CXCR4 in spinal dorsal horn. As expected, intrathecal pretreatment with the TLR_4_ antagonist, TAK-242 markedly ameliorated pain by inhibiting astrocytic and microglial activation, as shown by decreases in TLR_4_ immunoreactivity and the percentage of double-labeled cells. These protective effects were likely due in part to the reduced production of the downstream cytokines IL-1β and TNF-α, as well as for the recruitment of CXCL12 and CXCR4. Additionally, intrathecal pretreatment with CXCL12-neutralizing antibody and AMD3100 resulted in similar analgesic and anti-inflammatory effects as those receiving TAK-242 pretreatment. These results suggest that intrathecal blockade of CXCL12/CXCR4 expression may attenuate IR-induced pain sensation and the release of inflammatory cytokines by limiting glial TLR_4_ activation in spinal cord.

## Background

Spinal cord ischemia-reperfusion (IR) injury is a devastating, incapacitating complication that often inflicts persistent inflammatory pain and affects a considerable proportion of the human population worldwide [1]. Clinical treatment of hypoxia- or ischemia-induced inflammatory pain remains challenging due to the multifactorial and complex pathogenic mechanisms involved. Although pain is processed in neural networks, increasing evidence suggests that neuron-glial interactions, such as those of microglia and astrocytes, and Toll-like receptor (TLR)-mediated glial neuroinflammation in the spinal cord also play important roles in the promotion and maintenance of pain [2–4]. Among all TLR family members, TLR_4_ has been identified as a major mediator of inflammatory pain [5–7], with indirect effects most likely facilitated by glial or immune cells infiltrating into the injury or lesion [3]. As shown in our previous studies, IR-activated microglia, which exert their effects via TLR_4_ and induce further release of the proinflammatory chemokine IL-1β, can contribute to a secondary cascade of inflammatory responses as well as to decreased pain thresholds [8]. Additionally, a recent study showed that in a model of chronic pancreatitis-induced mechanical allodynia, TLR_4_ was greatly increased in astrocytes of the spinal dorsal horn. Further, our previous study suggested that neutralizing TLR_4_ function significantly attenuated the mechanical allodynia after spinal cord IR [8]. Although TLR_4_ expression is stable under normal physiological conditions, it can be quickly activated by several pathological stimuli. Finding and inhibiting the mediators that evoke and amplify TLR_4_ expression might lead to relatively broad therapies for interrupting the inflammatory feedback and improving the inflammatory pain of patients with IR injury.

Chemokines are small (8–10 kDa) chemotactic cytokines of the immune system that are currently classified into four families: the C, CC, CXC, and CX3C families [9]. The C-X-C chemokine ligand 12 (CXCL12), also known as SDF-1, belongs to the CXC family and is ubiquitously expressed in various cell types of the central nervous system [10]. The main function of CXCL12 is to activate immune cells (e.g., monocytes and macrophages) and attract them to inflammatory lesions by interacting with transmembrane G-protein-coupled receptors, such as C-X-C chemokine receptor 4 (CXCR4) [10, 11]. Increasing evidence suggests that CXCL12/CXCR4 expression not only is greatly promoted in areas of acute hypoxia and in ischemic lesions but also can attract stem cells to those areas [12,13]. In this context, CXCL12/CXCR4 expression has also been implicated in glutamate exocytosis and the production of tumor necrosis factor (TNF)-α, nuclear factor kappa-B (NF-κB) and interleukin (IL)-6 from glial cells, resulting in nociceptive sensitization [14–16]. This observation indicated that the CXCL12/CXCR4 expression might also modulate inflammatory pain through directing the activation and chemotaxis of microglia and astrocytes via their membrane-bound TLR_4_ receptors in hypoxic or ischemic nervous systems. Thus, in the present study, we examined the expression of CXCL12/CXCR4 and of glial TLR_4_ in a model of IR-induced inflammatory pain. Then, we investigated whether blocking CXCL12/CXCR4 or TLR_4_ via intrathecal injection of selective inhibitors could attenuate pain-related behavior and microglial and astrocytic activation.

## Materials and Methods

### Experimental animals and ethics statement

Sprague-Dawley (SD) rats weighing 200–250 g were purchased from the Experimental Animal Center of the China Medical University (Shenyang, China). The rats were housed in standard cages and maintained at 23°C with 40–50% humidity under a 12-h light/dark cycle. They were allowed free access to food and water for adaptive feeding for 1 week. Animal surgery was performed under anesthesia to minimize suffering, and experiments were approved by the Animal Care and Use Committee of the China Medical University.

We monitored the experimental rats everyday according to the amount of food and water they took, rectal temperature, and normal neurological signs (whether existing lower extremity sensory and motor dysfunction). Intravenous administration of an overdose of pentobarbital or delivering sevoflurane via precision vaporizer was the method of euthanasia for all animals utilized in this research.

### Rat model of post-ischemic pain induction

Spinal cord IR injury was induced using an aortic cross-clamping method, as previously described [17]. In brief, all rats were anesthetized with an intraperitoneal injection of 4% sodium pentobarbital at a dose of 50 mg/kg. Rectal temperature was monitored and maintained at 37.5 ± 0.5°C throughout the surgical procedure by using a heating lamp. The rats were placed in lateral position to expose the aortic arch through a cervicothoracic approach. Under direct visualization, the aortic arch was separated and cross-clamped between the left common carotid artery and the left subclavian artery. Ischemia was defined as a 90% decrease in femoral artery flow and was confirmed with a laser Doppler blood flow monitor (Moor Instruments, Axminster, Devon, UK). The artery clamps were opened after 8 min, followed by 72 h of reperfusion. Sham-operated rats underwent the same procedure without the cross clamping.

### Experimental protocol and drug delivery

A total of 120 rats were randomly assigned to the following groups via a random number table: sham, IR, IR + CXCL12-neutralizing antibody (IR + N), IR + control IgG (IR + C), antibody IR + CXCR4 antagonist (AMD3100, IR + A), and IR + TLR4 inhibitor (TAK-242, I/R + T).

In all groups, we used a microsyringe to intrathecally infuse 10 μl of normal saline, 20 μg/10 μl of CXCL12-neutralizing antibody (R&D Systems, USA), 10 μg/10 μl of control IgG antibody (R&D Systems, USA), 10 μg/10 μl of CXCR4-specific antagonist (AMD3100, EMD Millipore, USA) or 30 μg/10 μl of TAK-242 (EMD Millipore, USA). All drug doses were based on the results of preliminary experiments. Intrathecal infusions were administered for 5 consecutive days before surgery, and neurological signs were assessed after each injection. Only the rats with normal motor and sensory function were included in the follow-up experiments.

### Nociceptive behavioral testing

To quantify mechanical and thermal allodynia, the mechanical paw withdrawal threshold (PWT) and thermal paw withdrawal latency (PWL) of one hind-limb paw were assessed using von Frey filaments (Stoelting Co., Wood Dale, IL, USA) [18] and a hot plate (7370, UgoBasile Biological Research Apparatus Co., Ltd., Italy), as previously described [19]. Tests were performed by an observer blind to the experimental procedures before the surgery (baseline) and at 6 h intervals during the 48 h after surgery. PWT was defined as the pressure (g) at which the rat withdrew its paw. Filaments with logarithmically ascending or descending stiffness were applied once for 10 seconds to the plantar surface of the hind paw; this procedure was repeated four times with a minimum interval of 10 s to calculate the mean PWT.

PWL was measured 15 min after PWT was determined in the same rats. PWL was recorded as the time when the rat on the hot plate (50°C) clearly withdrew its paws. A maximum exposure time of 25 s was adopted to avoid tissue damage; measurements were obtained in triplicate with an interval of 10 min to assess the mean PWL. Mechanical and thermal allodynia were defined as a significant (*P* < 0.05) reduction in PWL and PWT, respectively, compared with the baseline.

### Western blots

After the behavioral tests were complete, the rats were anesthetized with an overdose of pentobarbital, and the L_4–6_ segments of the spinal cord were removed and processed for further biological analysis. After the samples were homogenized, cytoplasmic extracts were purified from each specimen using nucleoprotein and cytoplasmic protein extraction kits according to the manufacturer’s instructions (KGP-150; KangChen, Shanghai, China). Equivalent protein samples (50 μg) were separated using 10% SDS-PAGE, and the proteins were then transferred onto PVDF membranes (EMD Millipore, USA). The primary antibodies used in this experiment were rabbit anti-CXCL12 (1:300; Abcam, USA), rabbit anti-CXCR4 (1:200; Abcam, USA), mouse anti-TLR4 (Abcam) and anti-mouse GAPDH (dilution 1:10,000, Abcam). The membranes were incubated with the primary antibodies overnight on a shaker at 4°C. Then, the proteins were detected with horseradish peroxidase-conjugated anti-mouse or anti-rabbit secondary antibodies (BioSS, Beijing, China) for 1 h and visualized using an enhanced chemiluminescence kit (Beyotime Biotechnology, China). Semi-quantitation of the scanned images was performed using Quantity One software (Bio-Rad Laboratories, Milan, Italy).

### Double immunofluorescence

In brief, the L_4-6_ segments of the spinal cords were postfixed in 4% paraformaldehyde for 2–4 h and then cryoprotected in 30% sucrose in phosphate buffer overnight at 4°C. Then, the spinal cords were sectioned into 10-μm-thick slices using a cryostat (Leica CM3050s, Leica Biosystems, USA). The sections were blocked with 10% bovine serum albumin (BSA) for 1 h at room temperature and incubated overnight at 4°C with the following primary antibodies: rabbit polyclonal anti-CXCL12 antibody (1:100, Abcam, USA), rabbit monoclonal anti-CXCR4 antibody (1:100, Abcam, USA), mouse monoclonal anti-TLR_4_ antibody (1:100, Abcam, USA), mouse monoclonal anti-glial fibrillary acidic protein (GFAP) antibody (1:500, Abcam, USA), rabbit polyclonal anti-GFAP antibody (1:500, Abcam, USA), mouse monoclonal anti-ionized calcium-binding adaptor molecule-1 (Iba-1) antibody (1:400, Abcam, USA), and rabbit monoclonal anti-GFAP antibody (1:400, Abcam, USA). Then, the sections were washed four times for 5–10 min each in PBS containing 10% BSA and 0.25% Triton X-100. The sections were then incubated with secondary antibodies, Alexa 488-conjugated donkey anti-rabbit IgG (1:500, Molecular Probes, USA) and Alexa 594-conjugated donkey anti-mouse IgG (1:500, Molecular Probes, USA), for 2 h at room temperature. After immunostaining, these sections were examined using a Leica TCS SP2 laser-scanning microscope (Leica Microsystems, Buffalo Grove, IL, USA) and imaged using the attached digital camera. Nonspecific staining was determined by omitting the primary antibody. The data are expressed as numbers of positive cells/area/spinal section ± standard deviation (SD).

### Measurement of IL-1β and TNF-α using ELISA

Spinal cord samples were collected, homogenized, and centrifuged. The IL-1β and TNF-α content was determined using an ELISA kit (R&D Systems, Minneapolis, MN, USA). According to the manufacturer’s instructions, absorbance (A) was quantified at λ = 450 nm within 30 min, and the wavelength correction was set to 540 or 570 nm. The concentration of IL-1β and TNF-α in each sample was calculated based on the standard curve and is expressed in pg/mg protein.

### Statistical analysis

All data were collected by investigators blinded to the experimental procedures. All data were calculated as the mean ± SD and analyzed using SPSS software (version 17.0, SPSS Inc., Chicago, IL, USA). The behavioral data collected over time among the groups were compared by two-way repeated-measures ANOVA followed by Bonferroni *post hoc* tests. The other data were analyzed using one-way ANOVA followed by Newman–Keuls *post hoc* analysis. A *P* value < 0.05 was considered significant.

## Results

### Changes in mechanical and thermal allodynia after IR-induced pain

**Fig 1:**
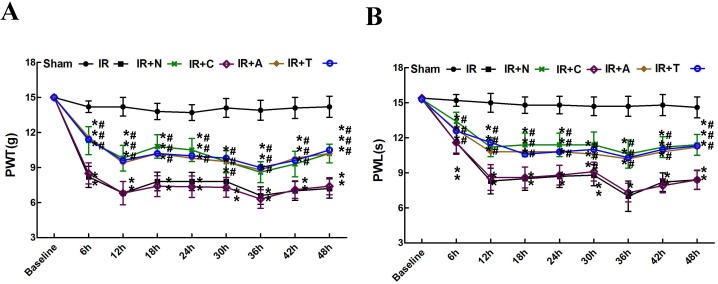
Changes in mechanical sensitivity to von Frey filaments and thermal allodynia to hot plate after IR. Compared with rats in the Sham group, significant decreases in PWT and PWL occurred in the IR groups during the 48 h post-injury period. Intrathecal injection of CXCL12-neutralizing antibody (I/R + N group), AMD3100 (I/R + A group) or TAK-242 (I/R + T group) 3 days before ischemia markedly increased PWT and PWL at all the observed time points. **P < 0.05 vs. the sham group; ## *P* < 0.05 vs. the IR group by two-way repeated-measures ANOVA followed by a Bonferroni post hoc test (n = 24 per group).

There were no significant differences in the baseline nociceptive thresholds among rats in the six groups (*P* > 0.05). As shown in Fig 1, the PWT and PWL of rats in the IR group significantly decreased relative to the baseline PWT and PWL in a time-dependent manner throughout the 48 h post-surgery period (*P*< 0.05); these results indicated the development and maintenance of IR-induced mechanical and thermal allodynia due to 8 min of aortic cross clamping. Meanwhile, no significant differences were observed between the IR and IR + C groups (*P* > 0.05). However, compared with rats in the IR group, PWT and PWL were markedly increased at all the observed time points in rats that were intrathecally injected with CXCL12-neutralizing antibody (I/R + N group), CXCR4-specific antagonist (I/R + A group) or TLR4 inhibitor (I/R + T group) (*P* < 0.05). There were no significant differences among the I/R + N, I/R + A and I/R + T groups at the above time points (*P* > 0.05).

### Changes in spinal CXCL12/CXCR4 and TLR4 expressions after IR-induced pain

**Fig 2:**
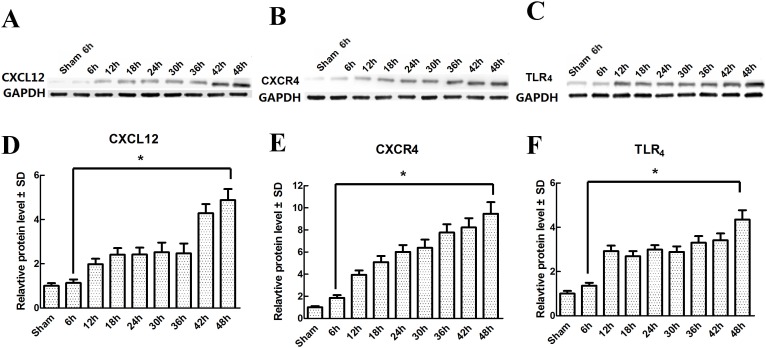
Changes in spinal CXCL12/CXCR4 and TLR_4_ expressions after IR-induced pain. (A-C) Representative immunoblot bands for CXCL12, CXCR4 and TLR_4_. GAPDH served as an internal standard. (D-F) Relative protein levels of CXCL12, CXCR4 and TLR_4_ were calculated as fold increases vs. the sham group. Data are expressed as the mean ± SD. *P < 0.05 vs. the sham group.

**Fig 3:**
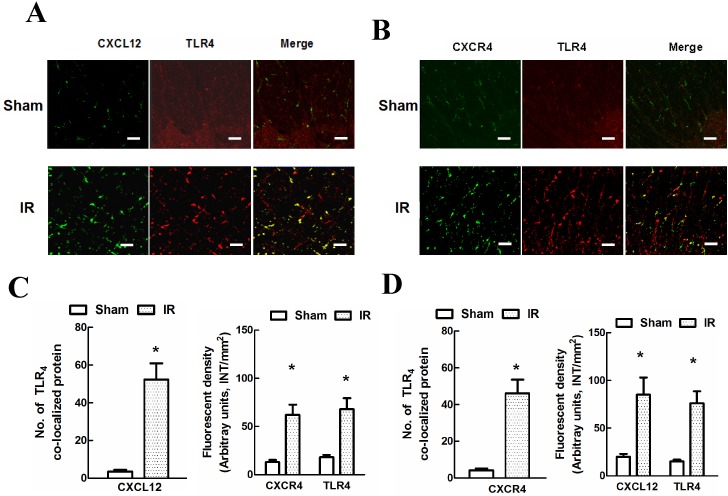
Localization of spinal CXCL12/CXCR4 and TLR_4_ expressions after IR-induced pain. (A-B) Representative micrographs showing the co-localization of CXCL12 (green) or CXCR4 (green) with TLR_4_ (red) at 48 h post-surgery. Scale bars represent 100 μm. (C-D) Quantification of the co-localized proteins (yellow signals) and the fluorescence density of each protein. Data are presented as the average of three measurements from each of three independent experiments. Double immunofluorescence showed that CXCL12/CXCR4 expression was highly co-localized with TLR_4_ expression after IR-induced pain. Data are expressed as the mean ± SD. *P < 0.05 vs. the sham group.

IR-induced chemokine changes are critical for glial cell activation [20]. As shown in Fig 2, the Western blot results showed that the protein levels of CXCL12, CXCR4 and TLR_4_ were very low at 48 h after the sham-operation. Compared with the sham group, IR induced time-dependent increases in the protein levels of CXCL12, CXCR4 and TLR_4_ over the course of 48 h post-injury (*P* < 0.05). The levels of these three proteins were increased at 6 h, had obviously increased further at 12 h, and peaked at 48 h. We also investigated CXCL12/CXCR4 expression involved in I/R-induced TLR_4_ activation using double immunofluorescence staining at 48 h post-injury. As shown in Fig 3A and 3B, strong CXCL12 and CXCR4 fluorescence signals were co-localized with the distribution of TLR_4_ in IR rats but not in sham-operated rats. A similar quantification of proteins co-localized with TLR_4_ (Fig 3C) and the immunoreactivity of each protein (Fig 3D) confirmed that CXCL12/CXCR4 expression was necessary for IR-induced TLR_4_ activation.

### Glial localization of CXCL12/CXCR4 expression in the spinal cord after IR-induced pain

**Fig 4:**
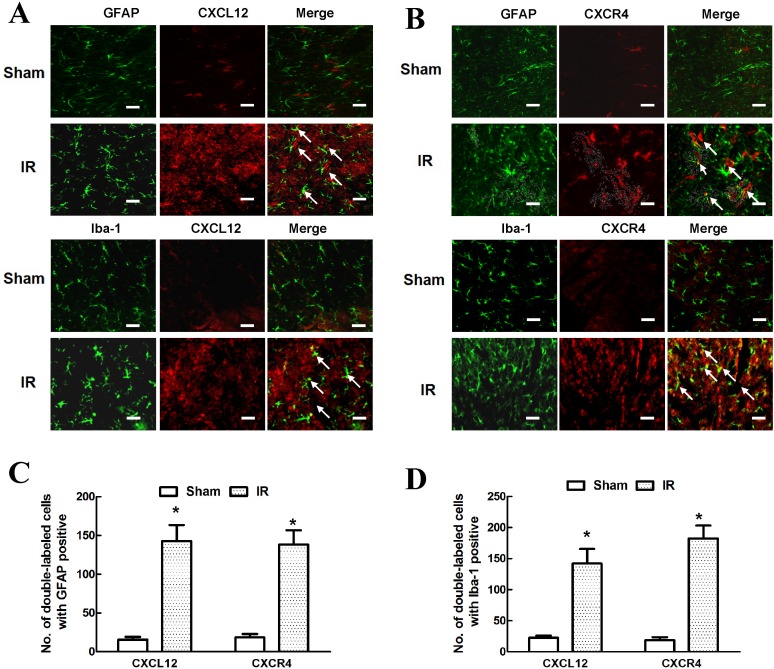
Glial localization of CXCL12/CXCR4 expression in the spinal cord after IR-induced pain. (A) Representative immunofluorescent localization of astrocytes (GFAP; green), microglia (Iba-1; green) and CXCL12 (red) in the spinal cords of the sham-operated or operated rats at 48 h post-surgery. (B) Representative immunofluorescent localization of astrocytes (GFAP; green), microglia (Iba-1; green) and CXCR4 (red) in the spinal cords of the sham-operated or operated rats at 48 h post-surgery. Scale bars represent 100 μm. (C) Quantification of double-labeled, GFAP-positive cells in the spinal cord. (D) Quantification of double-labeled, Iba-1-positive cells in the spinal cord. Double immunofluorescence showing that CXCL12/CXCR4 expression was up-regulated in IR-induced pain and highly co-localized with astrocytes and microglia. Data are expressed as the mean ± SD. *P < 0.05 vs. the sham group.

Glial cells are the main responders and effectors in the development and maintenance of neuropathic pain [20]. To further investigate the relationship between CXCL12/CXCR4 expression and activated glial cells, we performed double immunofluorescence staining for CXCL12 and CXCR4 with two major glial cell-specific markers, GFAP (for astrocytes) and Iba-1 (for microglia). As shown in Fig 4A and 4B, we observed widespread and strong GFAP and Iba-1 immunoreactivity in IR rats but not in sham-operated rats, indicating the activation of astrocytes and microglia during IR-induced pain (*P* < 0.05). In addition, fluorescent signals for CXCL12 in the same samples were not only very strong but also co-localized with the distribution of GFAP- and Iba-1-positive cells, suggesting that CXCL12 was induced by the astrocytes and microglia. Similarly, increased CXCR4 immunoreactivity was also localized predominantly on astrocytes and microglia. Quantification of the cells double-labeled with CXCL12 or CXCR4 and astrocyte or microglial markers confirmed that CXCL12/CXCR4 expression was up-regulated in IR-induced pain and that these signals were mainly expressed in astrocytes and microglia (Fig 4C and 4D).

### Blocking CXCL12/CXCR4 expression affects glial TLR4 and inflammatory cytokine release after IR-induced pain

**Fig 5:**
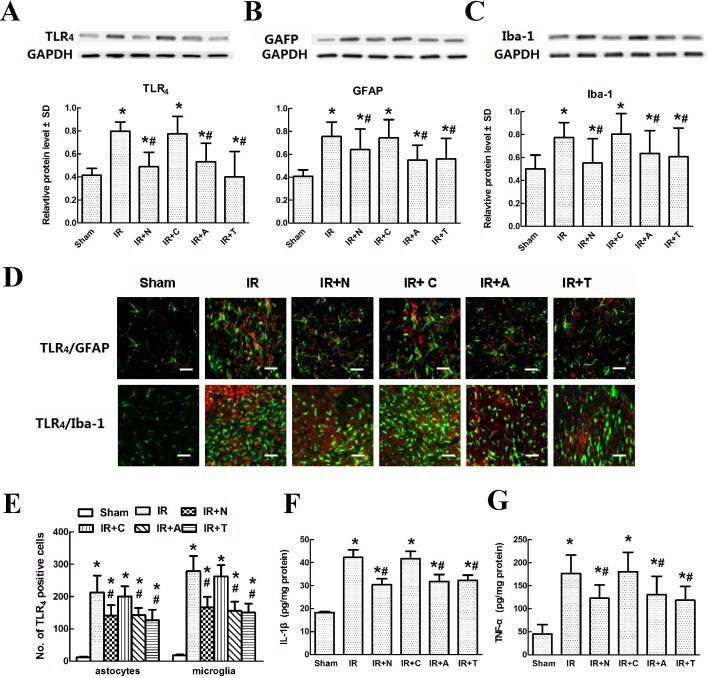
Effects of changes in spinal CXCL12/CXCR4 expression on glial TLR_4_ and inflammatory cytokine release after IR-induced pain. (A-C) Representative immunoblot bands for TLR_4_, GFAP and Iba-1. GAPDH served as an internal standard. Relative protein levels were calculated as fold increases vs. the sham group. Data are expressed as the mean ± SD. *P < 0.05 vs. the sham group; *^#^ *P* < 0.05 vs. the IR group. (D) Blocking CXCL12/CXCR4 expression in glial cells with co-localized TLR_4_ expression. Representative double-labeling images showing that intrathecal injection with CXCL12-neutralizing antibody, AMD3100 or TAK-242 significantly prevented microglia and astrocytes from up-regulating TLR_4_ at 48 h post-surgery. Scale bars represent 100 μm. (E) Quantification of TLR_4_-positive astrocytes or microglia in the spinal cord after IR-induced pain. (F-G) Blocking CXCL12/CXCR4 expression and inflammatory cytokine release after IR-induced pain. Quantification of IL-1β and TNF-α production in the spinal cord by ELISA. Data are expressed as the mean ± SD. *P < 0.05 vs. the sham group; *^#^ *P* < 0.05 vs. the IR group.

To test whether CXCL12/CXCR4 expression modulates astrocytic and microglial responses through the activation of their membrane-bound TLR_4_ and the release of IL-1β and TNF-α, we analyzed the levels of TLR_4_, Iba-1 and GFAP in injured spinal cords by Western blot and those of IL-1β and TNF-α by ELISA. Quantitative analysis of the Western blots showed that IR induced substantial increases in the levels of TLR_4_, Iba-1 and GFAP at 48 h post-injury compared with those in sham controls. As expected, intrathecal treatment with CXCL12-neutralizing antibody and AMD3100 produced similar and significant decreases in the expression levels of TLR_4_, Iba-1 and GFAP compared with those of rats intrathecally pretreated with TAK-242 (Fig 5A–5C, *P* < 0.05). Meanwhile, as shown in Fig 5D, confocal imaging indicated that many GFAP- and Iba-1-positive cells with identical, strong TLR4 immunoreactivity were activated in the injured spinal cords. In addition, the number of double-labeled glial cells was significantly decreased in rats pretreated with the CXCL12-neutralizing antibody, AMD3100 or TAK-242 (all *P* < 0.05). The quantitative data confirmed that IR-induced CXCL12/CXCR4 expression was involved in astrocytic and microglial activation via the surface TLR_4_ receptor (Fig 5E).

Finally, inflammatory cytokines downstream of IL-1β and TNF-α exhibited similar expression profiles in rats treated with neutralizing antibody, AMD3100 and TAK-242 (Fig 5F and 5G, *P* < 0.05), and there were no significant differences among these three groups (*P* > 0.05).

Furthermore, there were no significant differences between the IR group and the control IgG group in any of the above indices (*P* > 0.05).

## Discussion

Pain is a common clinical phenomenon that can be classified as inflammatory pain induced by tissue injury or neuropathic pain induced by nerve injury [21]. However, IR injury-induced pain is more complicated and is very difficult to treat because it involves both tissue and nerve injury in the context of temporary or permanent spinal cord ischemia [8, 22]. The present study aimed to further explore the underlying mechanisms identified in our previous study, in which we observed that spinal microglia were implicated in aberrant inflammatory cascades, which resulted in behavioral hypersensitivity, through the direct activation of TLR_4_ activation. Given the important role of glial TLRs in inflammatory pain, treatments targeting TLRs and inhibiting glial cell recruitment might offer increased therapeutic effectiveness for patients with IR injury. Increasing evidence suggests that chemokines, such as C-C motif ligand 2 (CCL2), CXCL10 and CXCL12, are involved in glial activation and that some of them contribute to pain processing [10, 23, 24]. In this study, we first characterized the protein expression and distribution of CXCL12 and CXCR4 in the spinal cord after 8 min of aortic arch occlusion. The increased CXCL12/CXCR4 expression observed was highly correlated with the expression of TLR_4_ over time, which was dependent on astrocytic and microglial activation, as indicated by Western blotting and double immunofluorescence staining. Furthermore, intrathecal antagonism of CXCL12/CXCR4 expression and pretreatment with TLR_4_ inhibitor produced similar effects regarding the alleviation of IR-induced hyperalgesia and cytokine release, indicating that the protective effects of blocking CXCL12/CXCR4 expression might be mediated by reduced glial TLR_4_ activation in spinal cord injury.

To date, an increasing amount of evidence has shown that CXCL12 plays an important role in neuropathic and inflammatory pain [10, 13, 16]. Previous studies have shown that CXCL12 mRNA and protein are both greatly up-regulated in the dorsal root ganglion (DRG) and in satellite glial cells in states of inflammatory pain [10, 25]. Chemokines exert their broad biological functions via their corresponding G-protein-coupled receptors; among those receptors, CXCR4 has been shown to respond strongly to CXCL12 [10, 12]. Upon coupling with CXCR4, CXCL12 has been shown to lead to rapid and sustained mechanical allodynia and thermal hypersensitivity through inflammatory cytokine up-regulation in the spinal cord after nerve injury [10, 16, 25]. In addition, in a rat model of spinal cord injury, prolonged CXCL12 immunoreactivity, co-expressed with pain-associated receptors (e.g., transient receptor potential vanilloid receptor-1, calcitonin gene-related peptide and TLR_4_) in the spinal contusion lesions, was reported to correlate with the development and maintenance of below-level pain [26]. In our study, the Western blot results showed that IR evoked time-dependent increases in the protein levels of CXCL12, CXCR4 and TLR_4_ over the course of the 48 h post-injury; the protein expression trends were similar, beginning as early as 6 h, clearly increasing further at 12 h, and peaking at 48 h. Furthermore, double immunofluorescence staining (Fig 2) confirmed that CXCL12/CXCR4 expression was necessary for IR-induced TLR_4_ activation, as identical TLR_4_ immunoreactivity was consistently co-localized with the distribution of CXCL12 and CXCR4 fluorescence.

The molecular mechanisms for the development and maintenance of inflammatory pain are multifactorial, and it is becoming clear that glial cell activation and networks (e.g., microglia and astrocytes) are critical for the initiation and maintenance of inflammatory pain [21]. Ischemia, hypoxia and inflammation can all induce astrocytic and microglial activation and proinflammatory cytokine and chemokine synthesis. In turn, the secretion of inflammatory chemokines can induce more astrocytic and microglial activation, as well as their recruitment into injured lesions, through the persistent activation of surface receptors for various signal pathways [1, 12, 14–16, 25]. Such positive feedback loops further facilitate glial-glial or even glial-neuronal networks and enhance nociceptive signal processing [21, 26]. Upon activation in various pain states, astrocytes exhibit hypertrophy and upregulate GFAP, whereas microglia positive for Iba-1 transformed from a ramified morphology to a rounded (amoeboid), macrophage-like shape, with thick and short processes [8, 16]. Furthermore, other immunolabeled slices with CD68- or ED1-positive labeling co-localized with Iba-1 expression suggested that CD68 and ED1 were more likely to be present on microglia capable of phagocytosis than on macrophages [27, 28]. In addition, strong CXCL12 immunoreactivity was reported in GFAP-positive astroglia and CD68/ED1-labeled inflammatory cells in the dorsal columns of the spinal cord [29]. Thus, it was not surprising to observe increased CXCL12 immunoreactivity expressed in both GFAP-positive astrocytes and Iba-1-positive microglia in our *in vivo* study. Similarly, increased CXCR4 immunoreactivity was clearly observed along with CXCL12 in the spinal dorsal horn, as well as in both astrocytes and microglia. These results suggested that CXCL12/CXCR4 expression contributed to inflammatory pain processing by regulating astrocytic-microglial activation. These results identify antagonizing CXCL12/CXCR4 expression and interrupting astrocytic-microglial activation as a potentially effective method for treating IR-related inflammatory pain.

TLRs are other well-known receptors that are widely expressed in various types of cells in the central nervous system [3]. Among various TLRs, glial TLR_4_ has been shown to be important in the evolution of pain processing by producing various proinflammatory mediators, including matrix metalloproteases (MMPs) and cyclooxygenases, the cytokines IL-1β and TNF-α, and the chemokines CCL2 and CXCL12 [4,6,8,24]. Given that the blockade of TLR_4_ activation alleviated inflammatory pain in our previous study [8], we further confirmed that CXCL12/CXCR4 expression contributes to the positive feedback loop of astrocytic and microglial activation via TLR_4_. We intrathecally injected specific antagonists of CXCL12, CXCR4 and TLR_4_ to evaluate changes in behavioral hypersensitivity and astrocytic and microglial TLR_4_ activation, as well as downstream IL-1β and TNF-α production. As expected, intrathecal injections of CXCL12-neutralizing antibody, AMD3100 and TAK-242 exerted similar analgesic effects on elevated PWT and PWL values in hind paws. After confirming the similar decreased protein expression levels of TLR_4_, GFAP and Iba-1, we also observed that the number of double immunofluorescence-labeled cells and the levels of downstream IL-1β and TNF-α changed in parallel. These results were consistent with those of a study by Lu DY, which suggested a mechanism linking CXCL12/CXCR4 expression with microglial cytokine production [15]. Thus, our data support the critical role of CXCL12/CXCR4 expression in the development and maintenance of IR-induced inflammatory pain via evoking astrocytic and microglial TLR_4_ positive feedback loops.

Notably, apart from CXCL12/CXCR4 expression in astrocytes and microglia, a recent study by Yang F showed that CXCL12/CXCR4 expression was also present in primary nociceptive neurons and that it contributed to persistent pain and hypersensitivity [10]. Given that primary sensory neurons located in the DRG are responsible for transferring peripheral stimuli to the brain through the spinal cord [10, 16, 26], it is possible that the neuronal-glial interaction could also contribute equally to pain processing, which merits further investigation. Finally, it is important to note that glial TLR4 activation is a double-edged sword, which can produce both beneficial and detrimental effects depending on the net effects of different chemokine signals and the complicated internal environments of *in vivo* experiments [8, 26, 30]. Because this study was an initial exploration of the inflammatory pain in spinal cord during IR, it is very possible that contradictory net effects will be observed under different experimental conditions. Thus, further combined efforts are required to better address how to fully maintain the intact beneficial effects of glial TLR_4_ activation and generate more effective clinical therapeutics.

## Conclusion

These findings suggest that spinal CXCL12/CXCR4 expression plays an important role in the development and maintenance of IR-induced inflammatory pain. Intrathecal suppression of CXCL12/CXCR4 expression successfully ameliorated pain sensation and cytokine release through the down-regulation of astrocytic and microglial TLR_4_ activation in the spinal cord.
